# Supplementary material for: Corneal confocal microscopy demonstrates sensory nerve loss in children with autism spectrum disorder
Source: PLoS One. 2023 Jul 12;18(7):e0288399. doi: 10.1371/journal.pone.0288399 (PMC10337936; doi:10.1371/journal.pone.0288399)
Supplement: Attachment — Submitted filename: ASD Rebutal Letter.docx [file pone.0288399.s001.docx]

Dear Editor,

We would like to express our gratitude to the reviewers for their constructive comments, which have greatly assisted us in enhancing our manuscript. Below, we have provided a point-by-point response to the comments, along with the revised manuscript highlighting the changes in red.

Journal requirements:

1-When submitting your revision, we need you to address these additional requirements.

Please ensure that your manuscript meets PLOS ONE's style requirements, including those for file naming. The PLOS ONE style templates can be found at <https://journals.plos.org/plosone/s/file?id=wjVg/PLOSOne_formatting_sample_main_body.pdf> and <https://journals.plos.org/plosone/s/file?id=ba62/PLOSOne_formatting_sample_title_authors_affiliations.pdf>.

Answer: Thank you. We have followed PLOS ONE STYLE requirements to the best of our knowledge.

2-We noticed you have some minor occurrence of overlapping text with the following previous publication(s), which needs to be addressed:

<https://doi.org/10.1002/ana.26484> <https://doi.org/10.1161/STROKEAHA.117.018289>

In your revision, ensure you cite all your sources (including your own works) and quote or rephrase any duplicated text outside the methods section. Further consideration is dependent on these concerns being addressed.

Answer: The first URL led us to a conference where the preliminary results of the study were presented as a Poster. In accordance with the policies of PLOS One, we would like to disclose that preliminary findings from this study were presented at the Annual Meeting of the American Neurological Association held on October 22-25, 2022, in Chicago, IL.

The second URL (<https://doi.org/10.1161/STROKEAHA.117.018289>) led us to our paper titled "Corneal Confocal Microscopy Detects Corneal Nerve Damage in Patients Admitted With Acute Ischemic Stroke." There is no overlap apart from text in the methods section which describes the same CCM methodology used for both studies.

3-Please provide additional details regarding participant consent. In the ethics statement in the Methods and online submission information, please ensure that you have specified what type you obtained (for instance, written or verbal, and if verbal, how it was documented and witnessed). If your study included minors, state whether you obtained consent from parents or guardians. If the need for consent was waived by the ethics committee, please include this information.

Answer: Thank you. We obtained written consent from the parents and also written assent from the children in their presence of their parents. This was approved by both Weill Cornell Medicine-Qatar and Hamad Medical Corporation IRBs. We have added the word "written" to the statement to provide more clarity. The revised sentence is as follows: Page 4, 2^nd^ Paragraph.

"All procedures were in accordance with the ethical standards of Weill Cornell Medicine-Qatar (IRB No: 19-00016) and Hamad Medical Corporation (MRC No: MRC-01-20-761) and the 1964 Helsinki declaration and its later amendments. Written informed consent was obtained from the parents or legal guardians, with the written assent of all the participants."

4-We note that the grant information you provided in the 'Funding Information' and 'Financial Disclosure' sections do not match. When you resubmit, please ensure that you provide the correct grant numbers for the awards you received for your study in the 'Funding Information' section.

Answer: Thank you. However, in the “Add a Funding Source:” the online system doesn't allow us to include our funding source from the Autism Research Institute - 4182 Adams Avenue, San Diego.

5-Thank you for stating the following in your Competing Interests section: "NO authors have competing interests". Please complete your Competing Interests on the online submission form to state any Competing Interests. If you have no competing interests, please state "The authors have declared that no competing interests exist", as detailed online in our guide for authors at <http://journals.plos.org/plosone/s/submit-now>. This information should be included in your cover letter; we will change the online submission form on your behalf.

Answer: Thank you. "The authors have declared that no competing interests exist."

6-Please note that in order to use the direct billing option, the corresponding author must be affiliated with the chosen institute. Please either amend your manuscript to change the affiliation or corresponding author or email us at [plosone@plos.org](mailto:plosone@plos.org) with a request to remove this option.

Answer: In the online system, Prof. Rayaz A Malik is the corresponding author, and the affiliated institute is documented as WCM-Q.

7-Please review your reference list to ensure that it is complete and correct. If you have cited papers that have been retracted, please include the rationale for doing so in the manuscript text, or remove these references and replace them with relevant current references. Any changes to the reference list should be mentioned in the rebuttal letter that accompanies your revised manuscript. If you need to cite a retracted article, indicate the article's retracted status in the References list and also include a citation and full reference for the retraction notice.

Answer: Thank You. We have carefully reviewed our reference list and found no retracted papers. Our reference list is complete and correct to the best of our knowledge.

Reviewers

Reviewer:#1: Summary
Overall, the paper is well and clearly written. This is an interesting study where authors for the first time demonstrate corneal nerve fibre loss in children with autism spectrum disorder (ASD).

Questions

It is interesting that no reduction in IWL was observed in children with ASD. One possible reason for this could be the fact that only 6 children had IW captured.

Answer: Thank You. We agree that with IW data from only 7 individuals may well have limited the power of the study. An alternate explanation is that lack of change in the IW reflects less distal nerve involvement and therefore provides insights into the underlying pathology of neurodegeneration in ASD. This differs from greater IW involvement in patients with diabetic neuropathy who typically have a distal dying back neuropathy. We have now expanded the discussion to clarify this on page 9.

“We show no loss of corneal nerves at the more distal inferior whorl which may indicate a unique pattern of neurodegeneration affecting proximal rather than distal nerves. This differs from our previous studies in adults with diabetic neuropathy showing greater loss of nerves at the inferior whorl compared to the central cornea, which is consistent with a dying back neuropathy [43].”

Were the IW images captured for 6 or 7 children? Figure 2E seems to show that there were 7 children with captured IW.

Answer: Thank you. We apologize for this error. The inferior whorl length was assessed in seven participants as per the figure. We have now corrected this in the text.

Have you considered quantifying Langerhans cells for the same images? It would be interesting to see if there are any differences between the two groups.

Answer: We agree that quantification of LC’s may provide insights into the underlying mechanisms of neurodegeneration in ASD. However, the variability in LC assessment if very large and with the small numbers we do not feel we would generate meaningful results. We are planning a larger study where we will quantify LC’s.

It might be beneficial for readers to add IW images as well, even if there was no significant difference between the two groups.

Answer: Thank you for your suggestion. We have now added IW images in Fig 1.

“Fig 1. Central corneal nerve image from a healthy control (A) and child with ASD (B) showing a reduction in main nerve fibers and branches (red arrows) in children with ASD and Inferior whorl (IW) image from a healthy control (C) and child with ASD (D) showing no difference.

Reviewer #2: The authors have demonstrated a reduction in corneal nerve fibers in children with autism spectrum disorder. This paper represents an important contribution to the literature as it is the first report of corneal nerve loss in ASD. The paper is very well-written, and the methodology is sound.

I have two minor comments: In the Methods section (page 4, line 24), the authors stated that the inferior whorl length parameter was available for six participants, which I believe should have been seven, as it appears in Figure 2E.

Answer: Thank you. We apologize for this error. The inferior whorl length was assessed in seven participants as per the figure 2E. We have now corrected this in the text.

Page 9, line 20; please edit as "... an alteration."

Answer: Thank you. We have edited the sentence accordingly.
